# FIT-based risk-stratification model effectively screens colorectal neoplasia and early-onset colorectal cancer in Chinese population: a nationwide multicenter prospective study

**DOI:** 10.1186/s13045-022-01378-1

**Published:** 2022-11-04

**Authors:** Shengbing Zhao, Shuling Wang, Peng Pan, Tian Xia, Rundong Wang, Quancai Cai, Xin Chang, Fan Yang, Lun Gu, Zixuan He, Jiayi Wu, Qianqian Meng, Tongchang Wang, Qiwen Fang, Xiaomei Mou, Honggang Yu, Jinghua Zheng, Cheng Bai, Yingbin Zou, Dongfeng Chen, Xiaoping Zou, Xu Ren, Leiming Xu, Ping Yao, Guangsu Xiong, Xu Shu, Tong Dang, Li Zhang, Wen Wang, Shengchao Kang, Hongfei Cao, Aixia Gong, Jun Li, Heng Zhang, Yiqi Du, Zhaoshen Li, Yu Bai, Gastrointestinal Early Cancer Prevention & Treatment Alliance of China (GECA)

**Affiliations:** 1 Department of Gastroenterology/Digestive Endoscopy Center, Changhai Hospital, Second Military Medical University/Naval Medical University, National Clinical Research Center for Digestive Diseases (Shanghai), National Quality Control Center of Digestive Endoscopy, Shanghai, 200433 China; 2 Department of Gastroenterology, Yantai Zhifu Hospital, Yantai, 264000 China; 3 Department of Gastroenterology, Renmin Hospital of Wuhan University, Wuhan, 430060 China; 4 Department of Gastroenterology, Yantaishan Hospital of Yantai City, Yantai, 264008 China; 5 Department of Gastroenterology, 967th Hospital of Joint Logistics Support Force, Dalian, 116021 China; 6 Department of Gastroenterology, Army Medical Center, Chongqing, 400042 China; 7 Department of Gastroenterology, Nanjing Drum Tower Hospital, The Affiliated Hospital of Nanjing University Medical School, Nanjing, 210008 China; 8 Digestive Disease Hospital of Heilongjiang Provincial Hospital, Harbin, 150001 China; 9 Department of Gastroenterology, Xinhua Hospital, Shanghai, Jiaotong University School of Medicine, Shanghai, 200092 China; 10 Department 1 of Gastroenterology, The First Affiliated Hospital of Xinjiang Medical University, Urumqi, 830054 China; 11 Department of Gastroenterology, Yueyang Hospital of Integrated Traditional Chinese and Western Medicine, Shanghai University of Traditional Chinese Medicine, Shanghai, 200083 China; 12 Department of Gastroenterology, The First Affiliated Hospital of Nanchang University, Nanchang, 330006 China; 13 Inner Mongolia Institute of Digestive Diseases, The Second Affiliated Hospital of Baotou Medical College, Inner Mongolia University of Science and Technology, Baotou, 014030 China; 14 Department of Gastroenterology, Beijing Rectum Hospital, Beijing, 100071 China; 15 Department of Gastroenterology, 900th Hospital of Joint Logistics Support Force, Fuzhou, 350025 China; 16 Department of Gastroenterology, 940th Hospital of Joint Logistics Support Force, Lanzhou, 730050 China; 17 Department of Gastroenterology, Affiliated Hospital of Chifeng University, Chifeng, 024099 China; 18 Department of Digestive Endoscopy, First Affiliated Hospital of Dalian Medical University, Dalian, 116011 China; 19 Department of Gastroenterology, The Second Affiliated Hospital of Kunming Medical University, Kunming, 650101 China; 20 Department of Gastroenterology, The Central Hospital of Wuhan, Tongji Medical College, Huazhong University of Science and Technology, Wuhan, 430014 China; 21 Department of Gastroenterology and Hepatology, Tongji Hospital, School of Medicine, Tongji University, Shanghai, 200092 China

**Keywords:** Risk-stratification model, Fecal immunochemical test, Early-onset colorectal cancer, Colorectal cancer screening

## Abstract

No fully validated risk-stratification strategies have been established in China where colonoscopies resources are limited. We aimed to develop and validate a fecal immunochemical test (FIT)-based risk-stratification model for colorectal neoplasia (CN); 10,164 individuals were recruited from 175 centers nationwide and were randomly allocated to the derivation (*n* = 6776) or validation cohort (*n* = 3388). Multivariate logistic analyses were performed to develop the National Colorectal Polyp Care (NCPC) score, which formed the risk-stratification model along with FIT. The NCPC score was developed from eight independent predicting factors and divided into three levels: low risk (LR 0–14), intermediate risk (IR 15–17), and high risk (HR 18–28). Individuals with IR or HR of NCPC score or FIT+ were classified as increased-risk individuals in the risk-stratification model and were recommended for colonoscopy. The IR/HR of NCPC score showed a higher prevalence of CNs (21.8%/32.8% vs. 11.0%, *P* < 0.001) and ACNs (4.3%/9.2% vs. 2.0%, *P* < 0.001) than LR, which was also confirmed in the validation cohort. Similar relative risks and predictive performances were demonstrated between non-specific gastrointestinal symptoms (NSGS) and asymptomatic cohort. The risk-stratification model identified 73.5% CN, 82.6% ACN, and 93.6% CRC when guiding 52.7% individuals to receive colonoscopy and identified 55.8% early-onset ACNs and 72.7% early-onset CRCs with only 25.6% young individuals receiving colonoscopy. The risk-stratification model showed a good risk-stratification ability for CN and early-onset CRCs in Chinese population, including individuals with NSGS and young age.

**Supplementary Information:**

The online version contains supplementary material available at 10.1186/s13045-022-01378-1.

**To the Editor,**

Risk-stratification screening efficiently reduces the incidence and mortality rate of colorectal cancer (CRC) [1], but no risk-stratification model has been extensively validated in China where the limited colonoscopy resources are mainly occupied by low-risk individuals with non-specific gastrointestinal symptoms (NSGS) [2, 3], who are considered equivalent to average-risk population for the risk of advanced colorectal neoplasia (ACN) [4, 5]. Current guidelines struggle to recommend to start colonoscopy screening at the age of 45 or 50 [6, 7]. However, any “one-size-fits-all” standard for age may prevent the detection of many early-onset CRCs [8, 9]. Herein, under the dilemma of inefficient detection, limited resources, and increasing early-onset CRC screening faced by colonoscopy practice, we developed and validated a risk-stratification model  for colorectal neoplasia (CN).

From 2018 to 2020, the National Colorectal Polyp Care (NCPC) program was implemented in 175 centers nationwide (Fig. 1A), where consecutive adult individuals who had no alarming symptoms or signs of CRC were enrolled, regardless of NSGS [4]. All participants completed questionnaires regarding baseline information and life risk factors and received fecal immunochemical tests (FITs) and colonoscopies. A central database was established to manage the uploaded data from all centers (accessed at www.ncrcgastro.org). The primary outcome was the CN [10]. The details of methods, including exclusion criteria, outcome measures, sample size calculation, and statistical analysis, are illustrated in Additional file 1: Supplementary Methods.

**Fig. 1:**
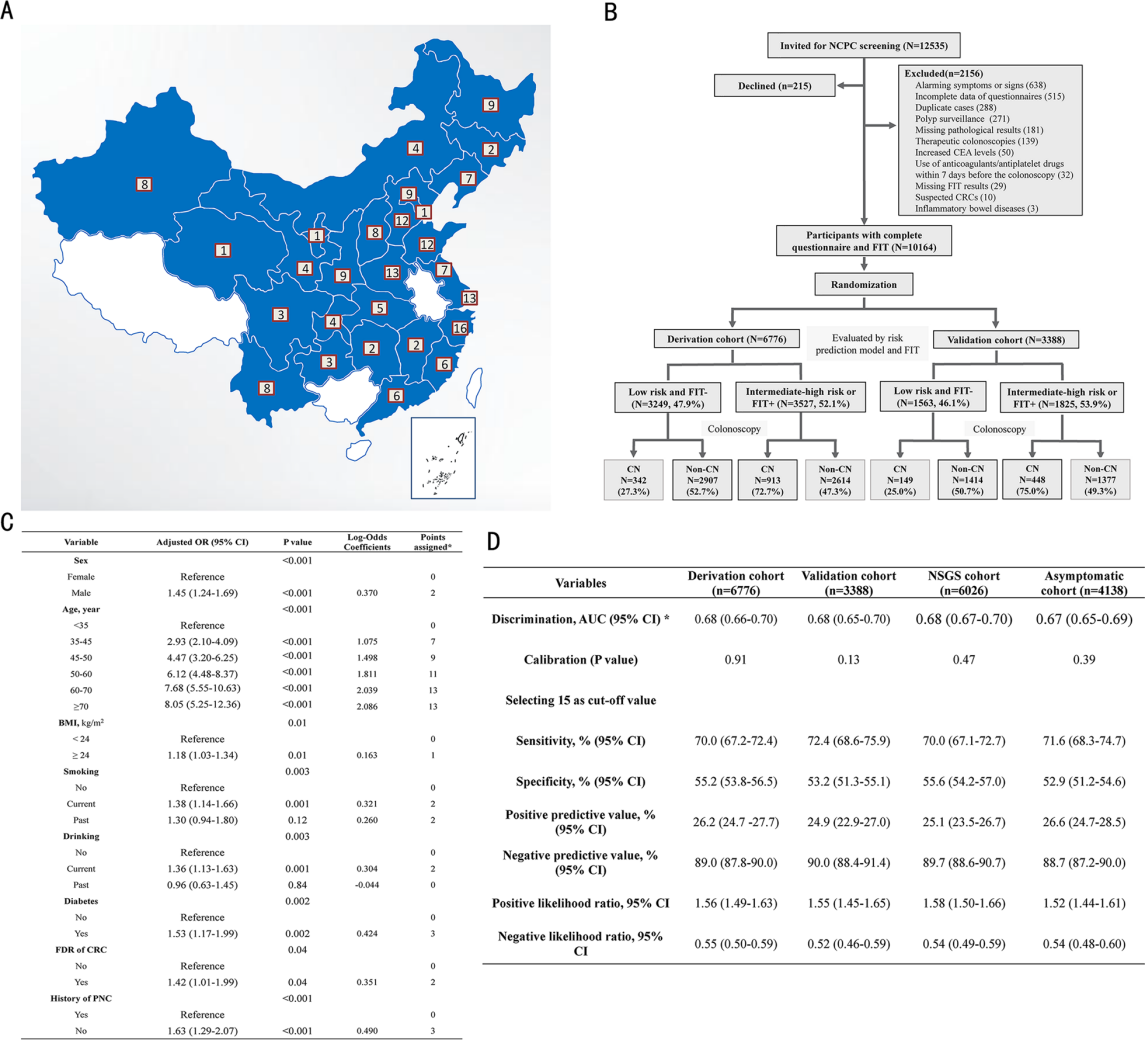
**A** The distribution of 175 participating centers in the provincial-level administrative regions of China. **B** Flowchart of enrollment, allocation, and study design. **C** Independent risk factors for colorectal neoplasia in the multivariate logistic regression model and points assigned to the NCPC score. * Points were assigned by dividing the Log-Odds coefficients by the absolute value of the smallest coefficient (BMI 0.163) and rounding up to the nearest integer. **D** Predicting performance of NCPC score in the derivation cohort, validation cohort, NSGS cohort, and asymptomatic cohort. * No significant differences were found for AUC between derivation and validation cohort (*P* = 0.80) or between NSGS and asymptomatic cohort (*P* = 0.31). NCPC, national colorectal polyp care; CEA, carcinoembryonic antigen; FIT, fecal immunochemical test; CRC, colorectal cancer; CN, colorectal neoplasia; OR, odds ratio; CI, confidence interval; BMI, body mass index; FDR, first-degree relative; PNC, previous negative colonoscopy; NSGS, non-specific gastrointestinal symptom; and AUC, area under the receiver operating characteristic curve

A total of 10,164 participants were enrolled (Fig. 1B), whose clinical characteristics were comparable between the derivation and validation cohort (Additional file 2: Tables S1–2). The univariate analysis identified 11 potential risk factors, and eight variables (sex, age, body mass index, smoking, drinking, diabetes, first-degree relative of CRC, history of previous negative colonoscopy) were identified as independent predicting factors for developing NCPC score (Additional file 2: Table S3, Fig. 1C), while the other variables were excluded (Additional file 2: Table S4). The NCPC score was divided into three levels according to the mean CN prevalence: low risk (LR 0–14, 0–17.4%), intermediate risk (IR 15–17, 18.8–24.0%), and high risk (HR 18–28, ≥ 25.9%) (Additional file 2: Table S5). Compared with FIT- individuals, FIT+ individuals showed higher risks for CN, ACN, and CRC in all subgroups of NCPC score (all *P* < 0.001) (Additional file 2: Table S6). Therefore, the risk-stratification model (Changhai Li’s Model) triaged individuals with IR or HR NCPC scores or FIT+ as increased-risk individuals to receive colonoscopy.

The model showed good calibrations no significant difference of area under curve (AUC) between the derivation and validation cohort (0.68 vs. 0.68, *P* = 0.80); consistent predicting performance in risk-stratification ability and individuals’ distribution were confirmed in the deviation and validation cohort (Figs. 1D, 2A). No significant difference of AUC was also found between NSGS and asymptomatic population (0.68 vs. 0.67, *P* = 0.31), where the predicting performances were demonstrated to be similar; individuals’ distribution and prevalence of CN and ACN were also found to be consistent between NSGS and asymptomatic individuals (Figs. 1D, 2B).

**Fig. 2:**
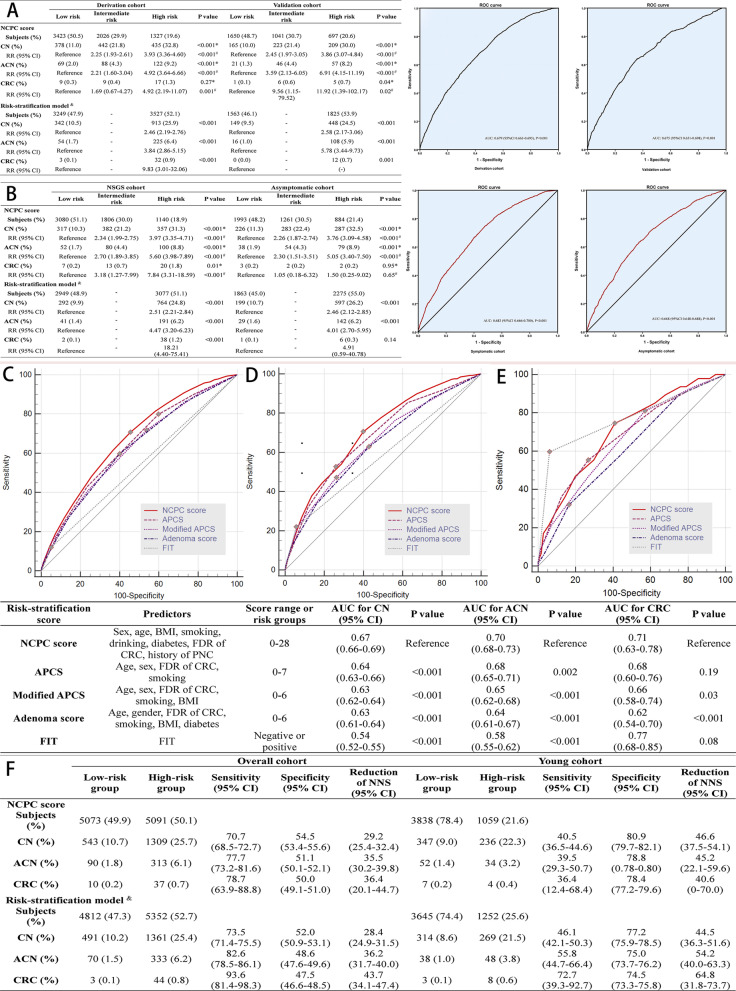
**A–B** Risk-stratification based comparisons of CN, ACN, and CRC prevalence between derivation and validation cohort or between NSGS and asymptomatic cohort. ^&^ Low risk represents participants with FIT- and low-risk score, and high risk represents participants with FIT+ or intermediate/high-risk score; * *P* value for intermediate risk vs. low risk; ^#^ *P* value for high risk vs. low risk. **C–E** Comparison of AUCs for CN, ACN, and CRC between the NCPC score and selected risk models or FIT for overall cohort. **F** Performance of NCPC score or risk-stratification model guided colonoscopy and estimated reduction of colonoscopy burden. ^&^ Low risk represents participants with FIT- and low-risk score, and high risk represents participants with FIT + or intermediate/high-risk score; reduction of NNS = (NNS by primary colonoscopy – NNS by NCPC (+FIT)-based algorithm)/(NNS by primary colonoscopy). AUC, area under the receiver operating characteristic curve; CN, colorectal neoplasia; CI, confidence interval; ACN, advanced colorectal neoplasia; CRC, colorectal cancer; NCPC, National Colorectal Polyp Care; BMI, body mass index; FDR, first-degree relative; PNC, previous negative colonoscopy; APCS, Asia–Pacific Colorectal Screening score; FIT, fecal immunochemical test; RR, relative risk. NNS, number needed for screening colonoscopy to detect one lesion; and ROC, receiver operating characteristic

Compared with FIT or other Asian models, the NCPC score showed the best discriminative ability for CN [0.67, *P* < 0.001] and ACN [0.70, *P* < 0.001 or = 0.002] [1, 11, 12] (Fig. 2C-E). The NCPC score could identify 70.7% CN, 77.7% ACN, and 78.7% CRC when reducing 29.2%, 35.5%, and 36.4% number needed for screening colonoscopies to detect one lesion (NNS), respectively (Fig. 2F). The risk-stratification model could identify 73.5% CN, 82.6% ACN, and 93.6% CRC when recommending 52.7% individuals to receive colonoscopy (Fig. 2F). By using risk-stratification model, only 25.6% young individuals will be recommended for colonoscopy, and 55.8% ACN and 72.7% CRC of young population could be identified when reducing 54.2% and 64.8% corresponding NNS, respectively (Fig. 2F).

In summary, a risk-stratification model (Changhai Li’s Model) for CN, consisting of FIT and NCPC score, was developed and validated to improve the efficiency of CRC screening. The model was able to save almost a half colonoscopy resources when maintaining a high sensitivity for CN, ACN, and CRC. Notably, 55.8% early-onset ACN and 72.7% early-onset CRC were identified with only 25.6% young individuals receiving colonoscopy. Consistent risk-stratifying performance was demonstrated between NSGS and asymptomatic population, which could rationally promote scope of CRC screening to cover the previously “ignored” NSGS population and avoid “indication gaming.” This model holds the promise as a feasible risk-stratification approach to improve the colonoscopy efficiency and CRC-screening scope in China and other countries with limited resources.

## Supplementary Information

**Additional file 1.** Supplementary methods

**Additional file 2.** **Table S1**. Clinical characteristics of participants and colonoscopy findings between the derivation and validation cohort; **Table S2**. Indications and quality indicators of colonoscopy between the derivation and validation cohort; **Table S3**. Univariate and multivariable analyses of variables included in the NCPC score based on the derivation cohort; **Table S4**. Univariate and multivariable analyses of variables excluded by the NCPC score based on derivation cohort; **Table S5**. Distribution of subjects, CN and ACN for each score category in the derivation and validation cohort; **Table S6**. CN risk stratified by NCPC score and FIT in the derivation and validation cohort.

**Additional file 3**. Supplementary lists

## Data Availability

All data related to the study are included in the paper and its supplementary materials.

## Abbreviations

ACN: Advanced colorectal neoplasia
AUC: Area under curve
CI: Confidence interval
CN: Colorectal neoplasia
CRC: Colorectal cancer
FIT: Fecal immunochemical test
HR: High risk
IR: Intermediate risk
LR: Low risk
NCPC: National Colorectal Polyp Care
NNS: Number needed for screening colonoscopy to detect one lesion
NSGS: Non-specific gastrointestinal symptom
