# Supplementary material for: FIT-based risk-stratification model effectively screens colorectal neoplasia and early-onset colorectal cancer in Chinese population: a nationwide multicenter prospective study
Source: J Hematol Oncol. 2022 Nov 4;15:162. doi: 10.1186/s13045-022-01378-1 (PMC9636700; doi:10.1186/s13045-022-01378-1)
Supplement: Supplementary file 1 — Additional file 1. Supplementary methods [file 13045_2022_1378_MOESM1_ESM.docx]

**FIT-based Risk-stratification Model Effectively Screens Colorectal Neoplasia and Early-Onset Colorectal Cancer in Chinese Population:**

**A Nationwide Multicenter Prospective Study**

**Supplementary Methods**

**Overview**

NCPC is a program initiated by the National Clinical Research Center for Digestive Disease (Shanghai, China) and Gastrointestinal Early Cancer Prevention & Treatment Alliance of China (GECA) to reduce the morbidity and mortality rates of gastrointestinal cancer through cancer screening[1]. It was consisted of questionnaire survey, FIT and colonoscopy and histological examination.

**Inclusion criteria**

Consecutive individuals aged 18-75 years who had no alarming symptoms or signs of CRC, regardless of intermittent non-specific gastrointestinal symptom (NSGS); individuals receiving colonoscopy for diagnosis; signed informed consent.

**Exclusion criteria**

Individuals with alarming gastrointestinal symptoms or signs, including hematochezia, melena, weight loss or anemia without specific causes, abdominal mass, and positive digital rectal examination; history of polypectomy or colonic resection; abnormal carcinoembryonic antigen levels; inflammatory bowel diseases; hereditary CRC syndromes; suspected or confirmed CRCs or other malignancies; intestinal obstruction; coagulopathy or use of anticoagulants/antiplatelet drugs within seven days before colonoscopy; therapeutic colonoscopies; severe chronic comorbidities; pregnancy or lactation.

**Rejection criteria**

Withdrawal of consent; severe complications during colonoscopy such as bleeding or perforation requiring surgical intervention.

**Outcome measures**

The primary outcome was CN, including CRCs, adenomas, sessile serrated lesions (SSLs), traditional serrated adenomas, and hyperplastic polyps ≥10 mm, which were recommended to undergo a shorter surveillance interval[2]. The secondary outcomes included CRC and ACN, which included CRCs, tubulovillous or villous adenomas, adenoma ≥10 mm or with high-grade dysplasia, SSLs ≥10 mm or with high-grade dysplasia and traditional serrated adenomas. The most advanced lesion was chosen with multiple lesions presented.

**Sample size calculation**

Based on the Chinese CN prevalence (14%)[3], the number of CNs per risk factor was set to be 20[4]. The 27 risk factors analyzed included sex, age, body mass index (BMI), FIT, smoking, drinking, diabetes, first-degree relatives (FDR) of CRC or colorectal adenomas, history of previous negative colonoscopy (PNC), living area, estrogen replacement therapy, exercise time, history of cholecystectomy, use of aspirin or non-steroidal anti-inflammatory drugs (NSAIDs) and folic acid, colonoscopy indications, and specific symptoms (no symptoms, change in bowel habit, constipation, diarrhea, loose stools, increased stool frequency; abdominal discomfort, abdominal pain, abdominal distension; non-specific symptoms etc.). Therefore, a minimal sample size of 3858 participants was required. The actual derivation cohort (6776) was large enough for the sufficient statistical power to further reduce bias of parameter estimates, decrease the risk of overestimation and underestimation [5] and improve the predictive performance [6].

**Statistical analysis**

A total of 10,164 participants were randomly divided into the derivation cohort (2/3, n=6776) or the validation cohort (1/3, n=3388). Each participant at the participating centers represented one unit of randomization and had the same chance of being selected, as previously described[7]. Univariate logistic regression was used to determine the relationship between CN and each potential risk factor in the derivation cohort. Any variable with P < 0.1 in univariate analysis was included in the multivariate logistic regression model[1]. The variables that retained statistical significance for CN in multivariate analyses were selected to develop the risk score. A regression coefficient-based scoring method was used (dividing the log-odds coefficients by the absolute value of the smallest coefficient) and points were rounded to the nearest integer for the score [7]. Compared with the mean prevalence of CN, the score was divided into low risk (LR), intermediate risk (IR) or high risk (HR). The receiver operating characteristic (ROC) curve and area under the ROC curve (AUC) was compared, with P value calculated by the method of Hanley[8, 9], while the 95% confidence intervals for proportions were calculated according to the efficient-score method[10]. Model calibration was evaluated by the Hosmer-Lemeshow χ^2^ statistic, and P value > 0.05 indicated good calibration. The discrimination ability was compared between the different cohorts, where the prevalence of CN and ACN for each score were also calculated; A two-sided P value < 0.05 was statistically significant. All statistical analyses were performed using IBM SPSS Statistics software v22 (Chicago, IL, USA) or R software (Shanghai, China).

**Questionnaire survey**

The self-reported questionnaire was previously validated [1, 7] and incorporated into a customized tablet computer, and included the following items: inclusion and exclusion criteria, baseline characteristics, life styles factors, clinical characteristics, history of previous negative colonoscopy (PNC), indications or symptoms for colonoscopy, and family history of CRC or colorectal adenomas among first-degree relatives (FDRs). Although eating habits were included in our previous model[7], they were not included in NCPC due to the difficulty in defining standard of eating habits nationwide and the limited supporting evidence[11, 12].

FIT

The InSure FIT (Enterix, Edison, NJ) was used[13]. Examinees were instructed to open the blue cap from the collection tube, insert the spiral stick into unhydrated stool at three different sites, return the spiral stick to the collection tube, and blend the content with the buffer supplied in the tube. Finally, an immunochromatographic test strip was inserted into the collection tube by either laboratory staff or the participant of the test. The cut-off level for positivity was set at ≥100 ng hemoglobin /mL buffer (corresponding to ≥20 μg hemoglobin /g feces).

Colonoscopy and histological examination

≥3 L polyethylene glycol was required to be used for bowel preparation and colonoscopies were performed by the colonoscopists with ≥1000 diagnostic procedures. The withdrawal time was required to be ≥6 minutes (excluding time for biopsy or polypectomy). After biopsy or resection, all lesions reported were confirmed by histological examination according to the guidelines of the 4^th^ World Health Organization Classification of Tumors. Disagreements were resolved through discussion with the experts of leading hospital.

**Data quality control**

The questionnaire survey, FIT, colonoscopy, and histological examination were performed by different investigators who were blinded to the other sections. GECA arranged training and quality control forums for participating hospitals in every province. The data completeness was automatically monitored by the database, while supervisors of leading center monitored the data accuracy weekly with supporting materials (colonoscopy and histological examination reports) refenced. After discussion with investigators of leading center, all qualified data were included.

**References**

1. Cai Q, Zhu C, Yuan Y, Feng Q, Feng Y, Hao Y, et al. Development and validation of a prediction rule for estimating gastric cancer risk in the Chinese high-risk population: a nationwide multicentre study. Gut. 2019;68(9):1576-87. doi: 10.1136/gutjnl-2018-317556. PubMed PMID: 30926654.

2. Gupta S, Lieberman D, Anderson JC, Burke CA, Dominitz JA, Kaltenbach T, et al. Recommendations for Follow-Up After Colonoscopy and Polypectomy: A Consensus Update by the US Multi-Society Task Force on Colorectal Cancer. Gastroenterology. 2020;158(4). doi: 10.1053/j.gastro.2019.10.026. PubMed PMID: 32044092.

3. Chen H, Li N, Ren J, Feng X, Lyu Z, Wei L, et al. Participation and yield of a population-based colorectal cancer screening programme in China. Gut. 2019;68(8):1450-7. doi: 10.1136/gutjnl-2018-317124. PubMed PMID: 30377193.

4. Ogundimu EO, Altman DG, Collins GS. Adequate sample size for developing prediction models is not simply related to events per variable. J Clin Epidemiol. 2016;76:175-82. doi: 10.1016/j.jclinepi.2016.02.031. PubMed PMID: 26964707.

5. Peduzzi P, Concato J, Kemper E, Holford TR, Feinstein AR. A simulation study of the number of events per variable in logistic regression analysis. J Clin Epidemiol. 1996;49(12):1373-9. Epub 1996/12/01. doi: 10.1016/s0895-4356(96)00236-3. PubMed PMID: 8970487.

6. Wynants L, Bouwmeester W, Moons KGM, Moerbeek M, Timmerman D, Van Huffel S, et al. A simulation study of sample size demonstrated the importance of the number of events per variable to develop prediction models in clustered data. J Clin Epidemiol. 2015;68(12):1406-14. doi: 10.1016/j.jclinepi.2015.02.002. PubMed PMID: 25817942.

7. Cai Q-C, Yu E-D, Xiao Y, Bai W-Y, Chen X, He L-P, et al. Derivation and validation of a prediction rule for estimating advanced colorectal neoplasm risk in average-risk Chinese. Am J Epidemiol. 2012;175(6):584-93. doi: 10.1093/aje/kwr337. PubMed PMID: 22328705.

8. Hanley JA, McNeil BJ. The meaning and use of the area under a receiver operating characteristic (ROC) curve. Radiology. 1982;143(1):29-36. PubMed PMID: 7063747.

9. Hanley JA, McNeil BJ. A method of comparing the areas under receiver operating characteristic curves derived from the same cases. Radiology. 1983;148(3):839-43. PubMed PMID: 6878708.

10. Newcombe RG. Two-sided confidence intervals for the single proportion: comparison of seven methods. Stat Med. 1998;17(8):857-72. Epub 1998/05/22. doi: 10.1002/(sici)1097-0258(19980430)17:8<857::aid-sim777>3.0.co;2-e. PubMed PMID: 9595616.

11. Moazzen S, van der Sloot KWJ, Bock GHd, Alizadeh BZ. Systematic review and meta-analysis of diet quality and colorectal cancer risk: is the evidence of sufficient quality to develop recommendations? Critical reviews in food science and nutrition. 2021;61(16):2773-82. doi: 10.1080/10408398.2020.1786353. PubMed PMID: 32613845.

12. Han MA, Zeraatkar D, Guyatt GH, Vernooij RWM, El Dib R, Zhang Y, et al. Reduction of Red and Processed Meat Intake and Cancer Mortality and Incidence: A Systematic Review and Meta-analysis of Cohort Studies. Annals of internal medicine. 2019;171(10):711-20. doi: 10.7326/M19-0699. PubMed PMID: 31569214.

13. Shapiro JA, Bobo JK, Church TR, Rex DK, Chovnick G, Thompson TD, et al. A Comparison of Fecal Immunochemical and High-Sensitivity Guaiac Tests for Colorectal Cancer Screening. Am J Gastroenterol. 2017;112(11):1728-35. doi: 10.1038/ajg.2017.285. PubMed PMID: 29016558.
