# Supplementary material for: FIT-based risk-stratification model effectively screens colorectal neoplasia and early-onset colorectal cancer in Chinese population: a nationwide multicenter prospective study
Source: J Hematol Oncol. 2022 Nov 4;15:162. doi: 10.1186/s13045-022-01378-1 (PMC9636700; doi:10.1186/s13045-022-01378-1)
Supplement: Supplementary file 2 — Additional file 2. Table S1. Clinical characteristics of participants and colonoscopy findings between the derivation and validation cohort; Table S2. Indications and quality indicators of colonoscopy between the derivation and validation cohort; Table S3. Univariate and multivariable analyses of variables included in the NCPC score based on the derivation cohort; Table S4. Univariate and multivariable analyses of variables excluded by the NCPC score based on derivation cohort; Table S5. Distribution of subjects, CN and ACN for each score category in the derivation and validation cohort; Table S6. CN risk stratified by NCPC score and FIT in the derivation and validation cohort. [file 13045_2022_1378_MOESM2_ESM.docx]

**FIT-based Risk-stratification Model Effectively Screens Colorectal Neoplasia and Early-Onset Colorectal Cancer in Chinese Population:**

**A Nationwide Multicenter Prospective Study**

**Supplementary Tables**

**Table S1.** Clinical characteristics of participants and colonoscopy findings between the derivation and validation cohorts.

| **Variables** | **All subjects**  **(n=10,164)** | **Derivation cohort**  **(n=6776)** | **Validation cohort (n=3388)** |
| --- | --- | --- | --- |
| **Women, n (%)** | 5029 (49.5) | 3353 (49.5) | 1676 (49.5) |
| **Mean age (SD), year** | 49.1 (12.0) | 48.9(12.1) | 49.4(11.9) |
| **Age, n (%)** |  |  |  |
| <35 | 1429 (14.1) | 971 (14.3) | 458 (13.5) |
| 35-45 | 1988 (19.6) | 1342 (19.8) | 646 (19.1) |
| 45-50 | 1480 (14.6) | 1009 (14.9) | 471 (13.9) |
| 50-60 | 3146 (31.0) | 2052 (30.3) | 1094 (32.3) |
| 60-70 | 1788 (17.6) | 1190 (17.6) | 598 (17.7) |
| ≥70 | 333 (3.3) | 212 (3.1) | 121 (3.6) |
| **Mean BMI (SD), kg/m^2^** | 23.5 (3.1) | 23.5(3.1) | 23.6(3.1) |
| BMI ≥24 kg/m^2^, n (%) | 4293 (42.2) | 2825 (41.7) | 1468 (43.3) |
| **Urban, n (%)** | 7239 (71.2) | 4798 (70.8) | 2441 (72.0) |
| **Smoking, n (%)** |  |  |  |
| Never | 8016 (78.9) | 5339 (78.8) | 2677 (79.0) |
| Current | 1755 (17.3) | 1177 (17.4) | 578 (17.1) |
| Past | 393 (3.9) | 260 (3.8) | 133 (3.9) |
| **Drinking, n (%)** |  |  |  |
| Never | 8077 (79.5) | 5390 (79.5) | 2687 (79.3) |
| Current | 1840 (18.1) | 1227 (18.1) | 613 (18.1) |
| Past | 247 (2.4) | 159 (2.3) | 88 (2.6) |
| **Diabetes, n (%)** | 437 (4.3) | 289 (4.3) | 148 (4.4) |
| **Post-cholecystectomy, n (%)** | 244 (2.4) | 156 (2.3) | 88 (2.6) |
| **Use of aspirin or non-steroidal anti-inflammatory drugs, n (%)** | 168 (1.7) | 104 (1.5) | 64 (1.9) |
| **FDR of CRC, n (%)** | 316 (3.1) | 207 (3.1) | 109 (3.2) |
| **FDR of adenomas, n (%)** | 145 (1.4) | 86 (1.3) | 59 (1.7) |
| **History of PNC, n (%)** | 940 (9.2) | 635 (9.4) | 305 (9.0) |
| **Positive FIT, n (%)** | 634 (6.2) | 431 (6.4) | 203 (6.0) |
| **Colonoscopy findings, n (%)** |  |  |  |
| CRC | 47 (0.5) | 35 (0.5) | 12 (0.4) |
| Advanced adenomas | 341 (3.4) | 234 (3.5) | 107 (3.2) |
| Advanced neoplasia | 403 (4.0) | 279 (4.1) | 124 (3.7) |
| Non-Advanced adenomas | 1369 (13.5) | 921 (13.6) | 448 (13.2) |
| Colorectal neoplasia | 1852 (18.2) | 1255 (18.5) | 597 (17.6) |
| IBD | 68 (0.7) | 41 (0.6) | 27 (0.8) |

Note: SD, standard deviation; BMI, body mass index; FDR: first-degree relative; CRC, colorectal cancer; PNC, previous negative colonoscopy; FIT, fecal immunochemical test; IBD, inflammatory bowel disease.

**Table S2.** Indications and quality indicators of colonoscopy between the derivation and validation cohorts.

| **Variables** | **All subjects**  **(n=10164)** | **Derivation cohort**  **(n=6776)** | **Validation cohort (n=3388)** |
| --- | --- | --- | --- |
| **Colonoscopy indications, n (%)** |  |  |  |
| **Asymptomatic** | 4138 (40.7) | 2725 (40.2) | 1413 (41.7) |
| **Change in bowel habit** | 2458 (24.2) | 1661 (24.5) | 797 (23.5) |
| Constipation | 700 (6.9) | 458 (6.8) | 242 (7.1) |
| Diarrhea | 1174 (11.6) | 804 (11.9) | 370 (10.9) |
| Loose stools | 469 (4.6) | 325 (4.8) | 144 (4.3) |
| Increased stool frequency | 13 (0.1) | 9 (0.1) | 4 (0.1) |
| Non-specific change in bowel habit | 102 (1.0) | 65 (1.0) | 37 (1.1) |
| **Abdominal discomfort** | 3270 (32.2) | 2189 (32.3) | 1081 (31.9) |
| Abdominal pain | 2300 (22.6) | 1528 (22.6) | 772 (22.8) |
| Abdominal distension | 955 (9.4) | 651 (9.6) | 304 (9.0) |
| Non-specific abdominal discomfort | 15 (0.1) | 10 (0.1) | 5 (0.1) |
| **Non-specific symptoms** | 298 (2.9) | 201 (3.0) | 97 (2.9) |
| **Quality indicators** |  |  |  |
| **CIR, n (%)** | 10105 (99.4) | 6735 (99.4) | 3370 (99.5) |
| **CIT, min (SD)** | 6.6 (5.0) | 6.6 (5.0) | 6.6 (5.1) |
| **WT, min (SD)** | 9.4 (4.8) | 9.3 (4.7) | 9.4 (5.1) |
| Time of biopsy or polypectomy, min (SD) | 3.23 (4.3) | 3.28 (4.2) | 3.14 (4.5) |
| WT for inspection, min (SD) | 8.4 (3.4) | 8.4 (3.3) | 8.5 (3.7) |
| Mean NWT, min (SD) | 8.0 (2.9) | 8.0 (2.8) | 8.1 (3.1) |
| **BBPS, n (SD)** | 7.6 (1.3) | 7.6 (1.3) | 7.5 (1.3) |
| BBPS ≥6, n (%) | 9716 (96.2) | 6480 (96.2) | 3286 (96.0) |
| **Bubble scores, n (SD)** | 7.8 (1.5) | 7.9 (1.5) | 7.8 (1.4) |
| Bubble scores ≥6, n (%) | 9712 (96.1) | 6794 (96.2) | 3233 (95.9) |

Note: CIR, cecal intubation rate; CIT, cecal intubation time; SD, standard deviation; WT, withdrawal time; NWT, negative colonoscopy withdrawal time; BBPS, Boston bowel preparation scale

**Table S3.** Univariate and multivariable analyses of variables included in the NCPC score based on the derivation cohort.

| **Variables** | **Derivation (n=6776)** | **CN (n=1255)** | **non-CN (n=5521)** | **P value** | **Adjusted OR (95% CI)** | **P value** |
| --- | --- | --- | --- | --- | --- | --- |
| **Sex, n (%)** |  |  |  | < 0.001 |  | < 0.001 |
| Female | 3353 (49.5) | 504 (15.0) | 2849 (85.0) |  | Reference |  |
| Male | 3423 (50.5) | 751 (21.9) | 2672 (78.1) |  | 1.45 (1.24-1.69) | < 0.001 |
| **Age, n (%)** |  |  |  | <0.001 |  | < 0.001 |
| <35 | 971 (14.3) | 48 (4.9) | 923 (95.1) |  | Reference |  |
| 35-45 | 1342 (19.8) | 177 (13.2) | 1165 (86.8) |  | 2.93 (2.10-4.09) | < 0.001 |
| 45-50 | 1009 (14.9) | 187 (18.5) | 822 (81.5) |  | 4.47 (3.20-6.25) | < 0.001 |
| 50-60 | 2052 (30.3) | 470 (22.9) | 1582 (77.1) |  | 6.12 (4.48-8.37) | < 0.001 |
| 60-70 | 1190 (17.6) | 315 (26.5) | 875 (73.5) |  | 7.68 (5.55-10.63) | < 0.001 |
| ≥70 | 212 (3.1) | 58 (27.4) | 154 (72.6) |  | 8.06 (5.25-12.36) | < 0.001 |
| **BMI, n (%)** |  |  |  | < 0.001 |  | 0.01 |
| < 24 kg/m^2^ | 3951 (58.3) | 641 (16.2) | 3310 (83.8) |  | Reference |  |
| ≥ 24 kg/m^2^ | 2825 (41.7) | 614 (21.7) | 2211 (78.3) |  | 1.18 (1.03-1.34) | 0.01 |
| **Smoking, n (%)** |  |  |  | < 0.001 |  | 0.003 |
| No | 5339 (78.8) | 874 (16.4) | 4465 (83.6) |  | Reference |  |
| Current | 1177 (17.4) | 312 (26.5) | 865 (73.5) |  | 1.38 (1.14-1.66) | 0.001 |
| Past | 260 (3.8) | 69 (26.5) | 191 (73.5) |  | 1.30 (0.94-1.80) | 0.12 |
| **Drinking, n (%)** |  |  |  | < 0.001 |  | 0.003 |
| No | 5390 (79.5) | 898 (16.7) | 4492 (83.3) |  | Reference |  |
| Current | 1227 (18.1) | 321 (26.2) | 906 (73.8) |  | 1.36 (1.13-1.63) | 0.001 |
| Past | 159 (2.3) | 36 (22.6) | 123 (77.4) |  | 0.96 (0.63-1.45) | 0.84 |
| **Diabetes, n (%)** |  |  |  | < 0.001 |  | 0.002 |
| No | 6487 (95.7) | 1161 (17.9) | 5326 (82.1) |  | Reference |  |
| Yes | 289 (4.3) | 94 (32.5) | 195 (67.5) |  | 1.53 (1.17-1.99) | 0.002 |
| **FDR of CRC, n (%)** |  |  |  | 0.04 |  | 0.04 |
| No | 6569 (96.9) | 1204 (18.3) | 5365 (81.7) |  | Reference |  |
| Yes | 207 (3.1) | 51 (24.6) | 156 (75.4) |  | 1.42 (1.01-1.99) | 0.04 |
| **History of PNC, n (%)** |  |  |  | 0.004 |  | < 0.001 |
| Yes | 635 (9.4) | 91 (14.3) | 544 (85.7) |  | Reference |  |
| No | 6141 (90.6) | 1164 (19.0) | 4977 (81.0) |  | 1.63 (1.29-2.07) | < 0.001 |

Note: CN, colorectal neoplasia; OR, odds ratio; CI, confidence interval; BMI, body mass index; FDR, first-degree relative; CRC, colorectal cancer; PNC, previous negative colonoscopy.

**Table S4.** Univariate and multivariable analyses of variables excluded by the NCPC score based on derivation cohort.

| **Variables** | **Derivation cohort (n=6776)** | **CN (n=1256)** | **non-CN (n=5520)** | **P value*** | **Adjusted OR (95% CI)** | **P value** |
| --- | --- | --- | --- | --- | --- | --- |
| **Living area, n (%)** |  |  |  | 0.09 |  | 0.75 |
| Urban | 4798 (70.8) | 864 (18.0) | 3934 (82.0) |  | Reference |  |
| Countryside | 1978 (29.2) | 391 (19.8) | 1587 (80.2) |  | 0.98 (0.85-1.12) | 0.75 |
| **Estrogen replacement therapy, n (%)** |  |  |  | 0.32 |  |  |
| No | 6749 (99.6) | 1252 (18.6) | 5497 (81.4) |  |  |  |
| Yes | 27 (0.4) | 3 (11.1) | 24 (88.9) |  |  |  |
| **Exercise time in a week, n (%)** |  |  |  | 0.32 |  |  |
| < 1 hour | 1732 (25.6) | 323 (18.6) | 1409 (81.4) |  |  |  |
| 1-2 hour | 1211 (17.9) | 234 (19.3) | 977 (80.7) |  |  |  |
| 3-4 hour | 1059 (15.6) | 175 (16.5) | 884 (83.5) |  |  |  |
| >4 hour | 2774 (40.9) | 523 (18.5) | 2251 (81.5) |  |  |  |
| **Post-cholecystectomy, n (%)** |  |  |  | 0.39 |  |  |
| No | 6620 (97.7) | 1222 (18.5) | 5398 (81.5) |  |  |  |
| Yes | 156 (2.3) | 33 (21.2) | 123 (78.8) |  |  |  |
| **Use of aspirin or non-steroidal anti-inflammatory drugs, n (%)** |  |  |  | 0.03 |  | 0.58 |
| No | 6672 (98.5) | 1227 (18.4) | 5445 (81.6) |  | Reference |  |
| Yes | 104 (1.5) | 28 (26.9) | 76 (73.1) |  | 1.14 (0.72-1.79) | 0.58 |
| **Use of folic acid, n(%)** |  |  |  | 0.64 |  |  |
| No | 6693 (98.8) | 1238 (18.5) | 5455 (81.5) |  |  |  |
| Yes | 83 (1.2) | 17 (20.5) | 66 (79.5) |  |  |  |
| **FDR of adenoma, n (%)** |  |  |  | 0.77 |  |  |
| No | 6690 (98.7) | 1238 (18.5) | 5452 (81.5) |  |  |  |
| Yes | 86 (1.3) | 17 (19.8) | 69 (80.2) |  |  |  |
| **Colonoscopy indications, n (%)** |  |  |  |  |  |  |
| **No symptom** |  |  |  | 0.33 |  |  |
| No | 4051 (59.8) | 735 (18.1) | 3316 (81.9) |  |  |  |
| Yes | 2725 (40.2) | 520 (19.1) | 2205 (80.9) |  |  |  |
| **Change in bowel habit** |  |  |  | 0.13 |  |  |
| No | 5115 (75.5) | 968 (18.9) | 4147 (81.1) |  |  |  |
| Yes | 1661 (24.5) | 287 (17.3) | 1374 (82.7) |  |  |  |
| **Constipation** |  |  |  | 0.39 |  |  |
| No | 6318 (93.2) | 1177 (18.6) | 5141 (81.4) |  |  |  |
| Yes | 458 (6.8) | 78 (17.0) | 380 (83.0) |  |  |  |
| **Diarrhea** |  |  |  | 0.10 |  | 0.16 |
| No | 5972 (88.1) | 1123 (18.8) | 4849 (81.2) |  | Reference |  |
| Yes | 804 (11.9) | 132 (16.4) | 672 (83.6) |  | 0.86 (0.70-1.06) | 0.16 |
| **Loose** **stools** |  |  |  | 0.48 |  |  |
| No | 6451 (95.2) | 1190(18.4) | 5261 (81.6) |  |  |  |
| Yes | 325 (4.8) | 65 (20.0) | 260 (80.0) |  |  |  |
| **Increased stool frequency** |  |  |  | 0.89 |  |  |
| No | 6767 (99.9) | 1254 (18.5) | 5513 (81.5) |  |  |  |
| Yes | 9 (0.1) | 1 (11.1) | 8 (88.9) |  |  |  |
| **Non-specific change in bowel habit** |  |  |  | 0.74 |  |  |
| No | 6711 (99.0) | 1244 (18.5) | 5467 (81.5) |  |  |  |
| Yes | 65 (1.0) | 11 (16.9) | 54 (83.1) |  |  |  |
| **Abdominal discomfort** |  |  |  | 0.92 |  |  |
| No | 4587 (67.7) | 848 (18.5) | 3739 (81.5) |  |  |  |
| Yes | 2189 (32.3) | 407 (18.6) | 1782 (81.4) |  |  |  |
| **Abdominal pain** |  |  |  | 1 |  |  |
| No | 5248 (77.4) | 972 (18.5) | 4276 (81.5) |  |  |  |
| Yes | 1528 (22.6) | 283 (18.5) | 1245 (81.5) |  |  |  |
| **Abdominal distension** |  |  |  | 0.88 |  |  |
| No | 6125 (90.4) | 1133 (18.5) | 4992 (81.5) |  |  |  |
| Yes | 651 (9.6) | 122 (18.7) | 529 (81.3) |  |  |  |
| **Non-specific abdominal discomfort** |  |  |  | 1 |  |  |
| No | 6766 (99.9) | 1253 (18.5) | 5513 (81.5) |  |  |  |
| Yes | 10 (0.1) | 2 (20.0) | 8 (80.0) |  |  |  |
| **Non-specific symptoms** |  |  |  | 0.49 |  |  |
| No | 6575 (97.0) | 1214 (18.5) | 5361 (81.5) |  |  |  |
| Yes | 201 (3.0) | 41 (20.4) | 160 (79.6) |  |  |  |

Note: CN, colorectal neoplasia; OR, odds ratio; CI, confidence interval; FDR, first-degree relative; ; NCPC, National Colorectal Polyp Care.

**Table S5.** Distribution of subjects, CN and ACN for each score category in the derivation and validation cohorts.

| **Risk level** | **Derivation cohort (n=6776)** | | | **Validation cohort (n=3388)** | | |
| --- | --- | --- | --- | --- | --- | --- |
|  | **Subjects** | **With CN** | **With ACN** | **Subjects** | **With CN** | **With ACN** |
| **LR, n (%)** |  |  |  |  |  |  |
| 0 | 10 | 0 (0.0) | 0 (0.0) | 4 | 0 (0.0) | 0 (0.0) |
| 1 | 2 | 0 (0.0) | 0 (0.0) | 4 | 0 (0.0) | 0 (0.0) |
| 2 | 7 | 0 (0.0) | 0 (0.0) | 5 | 0 (0.0) | 0 (0.0) |
| 3 | 271 | 5 (1.8) | 2 (0.7) | 137 | 7 (5.1) | 1 (0.7) |
| 4 | 58 | 4 (6.9) | 0 (0.0) | 30 | 1 (3.3) | 0 (0.0) |
| 5 | 234 | 15 (6.4) | 2 (0.9) | 96 | 8 (8.3) | 0 (0.0) |
| 6 | 108 | 3 (2.8) | 0 (0.0) | 55 | 4 (7.3) | 1 (1.8) |
| 7 | 134 | 11 (8.2) | 3 (2.2) | 62 | 9 (14.5) | 1 (1.6) |
| 8 | 94 | 3 (3.2) | 0 (0.0) | 40 | 2 (5.0) | 0 (0.0) |
| 9 | 87 | 3 (3.4) | 1 (1.1) | 34 | 2 (5.9) | 0 (0.0) |
| 10 | 458 | 47 (10.3) | 9 (2.0) | 207 | 17 (8.2) | 2 (1.0) |
| 11 | 192 | 21 (10.9) | 2 (1.0) | 105 | 9 (8.6) | 3 (2.9) |
| 12 | 550 | 72 (13.1) | 12 (2.2) | 266 | 27 (10.2) | 2 (0.8) |
| 13 | 392 | 50 (12.8) | 12 (3.1) | 172 | 23 (13.4) | 2 (1.2) |
| 14 | 826 | 144 (17.4) | 26 (3.1) | 433 | 56 (12.9) | 9 (2.1) |
| **IR, n (%)** |  |  |  |  |  |  |
| 15 | 596 | 112 (18.8) | 21 (3.5) | 323 | 60 (18.6) | 8 (2.5) |
| 16 | 768 | 171 (22.3) | 38 (4.9) | 377 | 73 (19.4) | 28 (7.4) |
| 17 | 662 | 159 (24.0) | 29 (4.4) | 341 | 90 (26.4) | 10 (2.9) |
| **HR, n (%)** |  |  |  |  |  |  |
| 18 | 371 | 96 (25.9) | 19 (5.1) | 182 | 49 (26.9) | 8 (4.4) |
| 19 | 341 | 104 (30.5) | 27 (7.9) | 201 | 51 (25.4) | 17 (8.5) |
| 20 | 232 | 82 (35.3) | 27 (11.6) | 113 | 33 (29.2) | 8 (7.1) |
| 21 | 203 | 73 (36.0) | 26 (12.8) | 94 | 34 (36.2) | 9 (9.6) |
| 22 | 81 | 29 (35.8) | 7 (8.6) | 44 | 19 (43.2) | 8 (18.2) |
| 23 | 60 | 28 (46.7) | 10 (16.7) | 36 | 12 (33.3) | 4 (11.1) |
| 24 | 22 | 11 (50.0) | 4 (18.2) | 16 | 6 (37.5) | 2 (12.5) |
| 25 | 4 | 2 (50.0) | 1 (25.0) | 6 | 2 (33.3) | 0 (0.0) |
| 26 | 12 | 9 (75.0) | 0 (0.0) | 5 | 3 (60.0) | 1 (20.0) |
| 27 | 0 | 0 (-) | 0 (-) | 0 | 0 (-) | 0 (-) |
| 28 | 1 | 1 (100.0) | 1 (100.0) | 0 | 0 (-) | 0 (-) |
| **Overall, n (%)** | 6776 | 1255 (18.5) | 279 (4.1) | 3388 | 597 (17.6) | 124 (3.7) |

Note: CN, colorectal neoplasia; ACN, advanced colorectal neoplasia; LR, low risk; IR, intermediate risk; HR, high risk.

**Table S6.** CN risk stratified by NCPC score and FIT in the derivation and validation cohorts.

|  | **Low risk** | | | **Intermediate risk** | | | **High risk** | | |
| --- | --- | --- | --- | --- | --- | --- | --- | --- | --- |
|  | **FIT-** | **FIT+** | **P value** | **FIT-** | **FIT+** | **P value** | **FIT-** | **FIT+** | **P value** |
| **Derivation cohort** |  |  |  |  |  |  |  |  |  |
| **Subjects, n (%)** | 3249 (94.9) | 174 (5.1) |  | 1882 (92.9) | 144 (7.1) |  | 1214 (91.5) | 113 (8.5) |  |
| **CN (%)** | 342 (10.5) | 36 (20.7) | <0.001 | 388 (20.6) | 54 (37.5) | <0.001 | 370 (30.5) | 65 (57.5) | <0.001 |
| RR (95% CI) | 1.97 (1.45-2.67) | |  | 1.82 (1.45-2.29) | |  | 1.89 (1.58-2.26) | |  |
| **ACN (%)** | 54 (1.7) | 15 (8.6) | <0.001 | 67 (3.6) | 21 (14.6) | <0.001 | 94 (7.7) | 28 (24.8) | <0.001 |
| RR (95% CI) | 5.19 (2.99-9.00) | |  | 4.10 (2.59-6.49) | |  | 3.20 (2.20-4.66) | |  |
| **CRC (%)** | 3 (0.1) | 6 (3.4) | <0.001 | 3 (0.2) | 6 (4.2) | <0.001 | 6 (0.5) | 11 (9.7) | <0.001 |
| RR (95% CI) | 37.35 (9.42-148.07) | |  | 26.14 (6.61-103.43) | |  | 19.70 (7.42-52.26) | |  |
| **Validation cohort** |  |  |  |  |  |  |  |  |  |
| **Subjects, n (%)** | 1563 (94.7) | 87 (5.3) |  | 984 (94.5) | 57 (5.5) |  | 638 (91.5) | 59 (8.5) |  |
| **CN (%)** | 149 (9.5) | 16 (18.4) | 0.01 | 200 (20.3) | 23 (40.4) | <0.001 | 182 (28.5) | 27 (45.8) | 0.01 |
| RR (95% CI) | 1.93 (1.21-3.08) | |  | 1.99 (1.41-2.79) | |  | 1.60 (1.18-2.17) | |  |
| **ACN (%)** | 16 (1.0) | 5 (5.7) | 0.004 | 40 (4.1) | 6 (10.5) | 0.04 | 44 (6.9) | 13 (22.0) | <0.001 |
| RR (95% CI) | 5.61 (2.11-14.97) | |  | 2.59 (1.15-5.85) | |  | 3.20 (1.83-5.58) | |  |
| **CRC (%)** | 0 (0.0) | 1 (1.1) | <0.05 | 5 (0.5) | 1 (1.8) | 0.29 | 2 (0.3) | 3 (5.1) | 0.005 |
| RR (95% CI) | (-) | |  | 3.45 (0.41-29.06) | |  | 16.22 (2.77-95.15) | |  |

Note: FIT, fecal immunochemical test; CN, colorectal neoplasia; RR, relative risk; CI, confidence interval; ACN, advanced colorectal neoplasia; CRC, colorectal cancer; NPCP, National Colorectal Polyp Care.
