# Supplementary material for: FIT-based risk-stratification model effectively screens colorectal neoplasia and early-onset colorectal cancer in Chinese population: a nationwide multicenter prospective study
Source: J Hematol Oncol. 2022 Nov 4;15:162. doi: 10.1186/s13045-022-01378-1 (PMC9636700; doi:10.1186/s13045-022-01378-1)
Supplement: Supplementary file 3 — Additional file 3. Supplementary lists [file 13045_2022_1378_MOESM3_ESM.docx]

**FIT-based Risk-stratification Model Effectively Screens Colorectal Neoplasia and Early-Onset Colorectal Cancer in Chinese Population:**

**A Nationwide Multicenter Prospective Study**

**Other Participating Centers and Physicians**

We would like to thank the following other members of the national colorectal polyp care for enrolling their patients in this study: Yingjian Zhang, The First Affiliated Hospital and College of Clinical Medicine of Henan University of Science and Technology, Luoyang; Qiong Nan, The First Affiliated Hospital of Kunming Medical University and Yunnan Institute of Digestive Disease, Kunming; Yali Wang, Third People’s Hospital of Datong, Datong; Zhilei Wu, Jiansanjiang People’s Hospital, Jiamusi; Guodong Yang, Affiliated Hospital of North Sichuan Medical College, Nanchong; Aibo Ma, Kunming First People’s Hospital of Yunnan Province, Kunming; Bing Hu, Eastern Hepatobiliary Surgery Hospital, Shanghai; Xing Chen, The First Hospital of Shanxi Medical University, Taiyuan; Shengyun Zhou, Xingtai People's Hospital, Xingtai; Ning Dai, Sir Run Run Shaw Hospital, Hangzhou; Kun zhuang, Xi'an Central Hospital, Xi'an; Jie Xu, Traditional Chinese Medical Hospital Of Xinjiang Uygur Autonomous Region, Urumqi; Jinzhong Chen, the First Affiliated Hospital of Xiamen University, Xiamen; Hongwei Xu, Kunshan Traditional Chinese Medicine Hospital, Suzhou; Huanhai Xu, The People's Hospital of Yueqing, Wenzhou; Fubing Yu, The Second People's Hospital of Yunnan, Kunming; Huiqing Jiang, The Second Hospital of Hebei Medical University, Hebei; Hong Xu, the First Hospital of Jilin University, Changchun; Hong Liu, Beijing Shijitan Hospital Affliated to the Capital Medical University, Beijing; Lijuan Huo, The First Hospital of Shanxi Medical University, Taiyuan; Weimin Wu, The First People's Hospital of Zunyi, Zunyi; Jing Zhang, Dalian Municipal Central Hospital, Dalian; Youlin Yang, First Affiliated Hospital of Harbin Medical University, Harbin; Rong Lin, Union Hospital of Tongji Medical College, Wuhan; Xiang Wang, The Second Hospital of Lanzhou University, Lanzhou; Peng Cao, Weinan Central Hospital, Weinan; Fangyu Wang, Genaral Hospital of Eastern Theater Command, Nanjing; Qiu Zhao, Zhongnan Hospital of Wuhan University, No.; Wenzhu Dong, 401 Hospital of the Chinese People's Liberation Army , Qingdao; Jianqiu Sheng, Beijing Military General Hospital, Beijing ; LiIi Yuan, Shanxi Bethune Hospital, Taiyuan; Bing Hu, West China Hospital,Sichuan; Xiaofeng Zhang, Hangzhou First People's Hospital, Hangzhou; Mingli Wu, The Fourth Hospital of Hebei Medical University, Shijiazhuang; Jian Lu, Kongjiang Hospital of Yangpu District, Shanghai ; Mingjun Sun, First Affiliated Hospital, Shengyang; Jie Pan, Wenzhou Central Hospital, Wenzhou; Yuehan Zhang, Hebei General Hospital, Shijiazhuang; Huaxing Wu, The Cancer Hospital of Harbin Medical University, Harbin; Lixian Zhang, Harrison International Peace Hospital, Hengshui; Pengfei Hou, Puyagn Oilfield General Hospital, Puyang; Hongwei Xu, Shandong Provincial Hospital Affiliated to Shandong University, Jinan; Xiaomei Xin, People's Hospital of Changji Hui Autonomous Prefecture, Changji; Xuanfu Xu, Shidong Hospital of Shanghai, Shanghai; Haoran Zhang, Chifeng Municipal Hospital, Chifeng; Liping He, Fujian Provincial Hospital, Fuzhou; Xiuling Li, Henan Provincial People's Hospital, Zhengzhou; Beiping Zhang, Guangdong Provincial Hospital of Traditional Chinese Medicine, Guangzhou; Feng Ji, The First Affiliated Hospital of Zhejiang university, Zhejiang; Yuanping Gao, People's Hospital of Meishan City, Meishan; Xiuli Zuo, Qilu Hospital, Jinan; Weigang Chen, First Affiliated Hospital of School of Medicine of Shihezi University, Shihezi; Pengfei Liu, Affiliated Hospital of Yan'an University, Yan'an; Linheng Wang, Dong Fang Hospital, Beijing; Jun Liu, Subei People's Hospital, Yangzhou; Long Zou, Shangluo Central Hospital, Shangluo; Zhanguo Nie, General Hospital of the Xinjiang Military Region, Ürümqi; Qiang Guo, the First People's Hospital of Yunnan Provinve, Kunming; Qinsheng Zhang, Henan Province Hospital of TCM, Zhengzhou; Xinyong Jia, Shandong Provincial Qianfoshan Hospital, Jinan; De-An Tian, Tongji Hospital, Wuhan; Lin Lu, Linyi People's Hospital, Linyi; Liangbi Xu, The Affiliated Hospital of Guizhou Medical University, Guiyang; Ping Xu, Shanghai Songjiang Distict Central Hospital, Shanghai; Wei Wu, The Second Affiliated Hospital and Yuying Children's Hospital of Wenzhou Medical University, Wenzhou; Yuzheng Xue, Affiliated Hospital of Jiangnan University/The Third People's Hospital of Wuxi, Wuxi; Zengjun Li, Shandong First Medical University and Shandong Academy of Medical Sciences, Shandong; Jianwei Shen, Ningbo Medical Center Lihuili Hospital, Ningbo; Bingrong Liu, The First Affiliated Hospital of Zhengzhou University, Zhengzhou; Shaofeng Wang, Changzhi People's Hospital, Changzhi; Liang Rong, the Fifth Affiliated Hospital of Xinjiang Medical University, Urumqi; Lina Guo, General Hospital of Heilongjiang General Bureau of Agricultural Reclamation, Harbin; Chunmeng Jiang, the Second Affiliated Hospital of Dalian Medical University, Dalian; Yan Liu, the Fifth Clinical Center of Chinese PLA General Hospital (307 Hospital), Beijing; Side Liu, Nanfang Hospital, Guangdong; Shuchang Xu, Tongji Hospital, Shanghai; Yu Lan, Beijing Jishuitan Hospital, Beijing; Xu Ren, Heilongjiang Provincial Hospital, Harbin; Zhanxiong Xue, Second Affiliated Hospital of Wenzhou Medical University, Wenzhou; Jiang Wu, The Second Affiliated Hospital of Xinjiang Medical University, Urumqi; Zibin Tian, The Affiliated Hospital of Qingdao University, Qingdao; Yiyang Dai, The Fourth Affiliated Hospital of Zhejiang University School of Medicine, Yiwu; Yanjun Li, Chenzhou First People's Hospital, Chenzhou; Shuixiang He, The First Affiliated Hospital of Medical School of Xian Jiaotong University, Xi'an; Kunming Huang, Zibo Central Hospital, Zibo; Xiaojun Yang, Chongqing Hospital of Traditional Chinese Medicine, Chongqing; Rui Ji, The First Hospital of Lanzhou University, Lanzhou; Qingcai Wang, Taian City Central Hospital, Tai'an; Siyu Sun, Shengjing Hospital, Shengyang; Pinghong Zhou, Zhongshan Hospital, Shanghai; Baohong Xu, Beijing Luhe Hospital, Beijing; Yipin Liu, Yantai Affiliated Hospital of Binzhou Medical University, Yantai; Zhiyong Zhang, Tangshan Gongren Hospital, Tangshan ; Bin Lv, The First Affiliated Hospital of Zhejiang Chinese Medical University, Hangzhou; Tao Yu, Shaanxi Traditional Chinese Medicine Hospital, Xi'an; Weiqing Chen, Chongqing University Cancer Hospital, Chongqing; Zhuo Yang, General Hospital of Shenyang Military Region, Shenyang; Bianying Liu, Shanxi Coal Center Hospital, Taiyuan; Biguang Tuo, Affiliated Hospital of Zunyi Medical University, Zunyi; Zhenzhen Liu, The First Affiliated Hospital of Zhengzhou University, Zhengzhou; Zhongyuan Tao, No.1 People's Hospital of Dali City, Dali; Rui Jiang, Gansu Provincial Hospital, Lanzhou; Qiming Lu, The Second Affiliated Hospital, Jiaxing; Junping Wang, Shanxi Provincial People's Hospital, Shanxi; Guiquan Chen, Dongguan People's Hospital, Dongguan; Jinhai Wang, the Second Affiliated Hospital of Xian Jiaotong University, Xi'an; Nian Fang, The Third Affiliated Hospital of Nanchang University, Nanchang; Shaoqi Yang, General Hospital of Ningxia Medical University, Yinchuan; Xiaoyu Liu, Yulin Second Hospital, Yulin; Haifeng Jin, The 980th Hospital of the PLA Joint Logistics Support Force, Shijiazhuang; Duowu Zou, Ruijin Hospital, Shanghai; Xinying Wang, Zhujiang Hospital, Guangzhou; Suyun Zheng, Qujing First Hospital/Qujing Affiliated Hospital of Kunming Medical University, Qujing; Mingxin Zhang, The First Affiliated Hospital of Xi'an Medical University, Xi'an; Xiangchun Lin, Peking University International Hospital, Beijing; Zili Tian, Affiliated Hospital of Hebei University, Baoding; Yong Yu, The Fifth Affiliated Hospital of Zhengzhou University, Zhengzhou; Huili Wu, Zhengzhou Central Hospital, Zhengzhou; Junmin Wang, The Third Hospital of Hebei Medical University, Shijiazhuang; Ying Xiong, Baoding First Central Hospital, Baoding; Wen Li, Tianjin People's Hospital, Tianjin; Zhufeng Yang, Hebei Traditional Chinese Medicine Hospital, Shijiazhuang; Mingqing Zhang, Affiliated Southeast Hospital of Xiamen University, Xiamen; Yan Liu, Changxing People's Hospital, Huzhou; Suzuan Chen, The First Affiliated Hospital of Shantou University Medical College, Shantou; Bing Xia, Hospital of Traditional Chinese Medicine of Qiqihar, Qiqihar; Xiaocui Yang, Ankang Central Hospital, An'kang; Liang Wang, Cangzhou Central Hospital, Cangzhou; Aiming Yang, Peking Union Medical College Hospital, Beijing; Zhiming Suo, Huaihe Hospital of Henan University, Kaifeng; Feng Gao, People's Hospital of Xin jiang Uygur Autonomous Region, Urumqi; Wei Liang, Fujian Provincial Hospital, Fujian; Jiao Lv, No.411 Hospital of CPLA, Shanghai ; Jianting Cai, The Second Affiliated Hospital of Zhejiang University School of Medicine, Zhejiang; Lin Miao, Second Affiliated Hospital of Nanjing Medical University, Nanjing; Lifeng Fang, Zhengzhou First People's Hospital, Zhengzhou; Jinghuai Li, Mudanjiang City Hospital of TCM, Mudanjiang; Guangrong Zhao, Zhangjiajie City People's Hospital, Zhangjiajie; Xiaoyun Ding, Ningbo First Hospital, Ningbo; Feng Liu, The Shanghai Tenth People's Hospital, Shanghai; Linglong Xue, Sixth Hospital of Shanxi Medical University (General Hospital of TISCO) Taiyuan, Shanxi; Hongli Tian, Bayinguoleng Mongol Autonomous Prefecture People's Hospital, Xinjiang; Dongde Ji, Qinghai Provincial People's Hospital, Xining; Lang Lin, The First People's Hospital of Cangnan, Wenzhou; Huiming Tu, Affiliated Hospital of Jiangnan University/The Fourth People's Hospital of Wuxi, Wuxi; Hongfeng Han, Luoyang Central Hospital Affiliated to Zhengzhou University, Luoyang; Xinwei Wang, Nanyang Central Hospital, Nanyang; Shuqiang Dong, Yunnan Cancer Hospital /the Third Affiliated Hospital of Kunming Medical University, Kunming; Yang Xu, Daqing Oilfield General Hospital, Daqing; Junxi Wang, The First Affiliated Hospital of Fujian Medical University, Fuzhou; Liang Zhong, Hua Shan Hospital Affiliated to Fudan University, Shanghai; Tieli Peng, The Sixth Affiliated Hospital of Guangzhou Medical University, Qingyuan; Zhixin Yao, Siping Hospital of China Medical University, Siping; Liping Ye, Taizhou Hospital of Zhejiang Province, Taizhou; Song He, The Second Affiliated Hospital of Chongqing Medical University, Chongqing.
